# Wealth Disparities in End-of-Life Symptom Burden Among Older Adults

**DOI:** 10.1001/jamanetworkopen.2025.0201

**Published:** 2025-03-06

**Authors:** Irena Cenzer, Kenneth E. Covinsky, Sarah H. Cross, Claire K. Ankuda, Lauren J. Hunt, Melissa D. Aldridge, Krista L. Harrison

**Affiliations:** 1 Division of Geriatrics, University of California, San Francisco; 2 Veterans Affairs Medical Center, San Francisco, California; 3 Division of Palliative Medicine, Department of Family and Preventive Medicine, Emory University, Atlanta, Georgia; 4 Brookdale Department of Geriatrics and Palliative Medicine, Icahn School of Medicine, New York, New York; 5 Philip R. Lee Institute for Health Policy Studies, University of California, San Francisco; 6 Global Brain Health Institute, University of California, San Francisco; 7 Department of Physiological Nursing, University of California, San Francisco; 8 James J. Peters Bronx VA Medical Center, New York, New York

## Abstract

**Importance:**

Understanding how wealth influences the end-of-life experience is crucial for improving patient care and policy, particularly with regard to symptom burden.

**Objective:**

To examine the prevalence of 12 end-of-life symptoms by wealth categories; the association between wealth and a composite measure of high symptom burden; and whether this association is mediated by multimorbidity, functional impairment, or dementia.

**Design, Setting, and Participants:**

This cohort study used data from the Health and Retirement Study (HRS), a nationally representative survey of older adults in the US, on decedents who died between January 1, 2000, and February 28, 2021, from proxy-reported exit interviews through February 28, 2021. Decedents were HRS participants aged 65 years or older who had completed a core interview within 24 months before death and had a proxy-reported exit interview. The data analysis was performed from October 6, 2023, through November 26, 2024.

**Exposures:**

Wealth (assets opposed to income) was categorized into low (bottom quartile, <$6000), medium (middle 2 quartiles, $6000-$120 000), and high (top quartile, >$120 000).

**Main Outcomes and Measures:**

The presence of 12 individual end-of-life symptoms and a composite measure of high symptom burden (≥7 symptoms) were analyzed using modified Poisson regression. Mediators included multimorbidity, functional impairment, and dementia. Models were adjusted for age, sex, marital status, race and ethnicity, education, childhood socioeconomic status, HRS proxy respondent relationship, location of death, survey timing, year of death, and private insurance.

**Results:**

Of 8976 decedents (mean [SD] age, 81.3 [8.6] years; 4927 women [55.1%] women) in the cohort, 2197 (22.5%) were included in the low wealth category, 4534 (50.5%) in the medium wealth category, and 2245 (27.1%) in the high wealth category. Decedents with lower wealth had a higher prevalence of end-of-life symptoms. High symptom burden was most common in the low wealth group (800 decedents [37.0%]) vs the medium (1301 decedents [28.0%]) and high wealth (512 decedents [23.2%]) groups. Low wealth was associated with a significantly increased risk of high symptom burden vs medium wealth (adjusted risk ratio, 0.79; 95% CI, 0.73-0.87) and high wealth (adjusted risk ratio, 0.71; 95% CI, 0.63-0.79). Multimorbidity, functional impairment, and dementia were more common in the low wealth group and mediated this association, with functional impairment being the strongest mediator.

**Conclusions and Relevance:**

This cohort study found that lower wealth was associated with a higher symptom burden at the end of life, mediated in part by higher rates of multimorbidity, functional impairment, and dementia. These findings highlight the need for policies and programs to support patients with lower financial resources to improve end-of-life experiences and mitigate wealth disparities.

## Key Points

**Question:**

What is the association between wealth and end-of-life symptom burden, and what factors mediate symptom burden?

**Findings:**

In this cohort study of 8976 US older adults, low wealth was significantly associated with higher end-of-life symptom burden (ie, presence ≥7 of 12 symptoms). Decedents with low wealth were more likely to experience high symptom burden compared with those with medium or high wealth, with functional impairment, multimorbidity, and dementia mediating this association.

**Meaning:**

These findings suggest that addressing functional disabilities and providing better support at the end of life may help improve patients’ end-of-life experiences and reduce disparities associated with wealth.

## Teaser

This cohort study uses Health and Retirement Study data to examine the prevalence and mediating factors of end-of-life symptoms by wealth categories among US older adult decedents.

## Introduction

End of life among older adults (aged 65 years or older) is often characterized by serious illness, multimorbidity, functional disability, and cognitive decline. Many factors contribute to how a person and their family experience this period, including financial resources.

Income and wealth are 2 primary measures of financial resources. Income reflects current earnings and flow of resources, and wealth represents a set of resources (eg, real estate, stocks) accumulated over a lifetime.^1^ Both lower income and lower wealth are associated with a wide range of negative health outcomes throughout the life course, including shorter life expectancy^2^ and higher levels of disability.^3^ It is less clear whether financial resources are also associated with experience at the end of life. Existing research has predominantly focused on disparities in quality of end-of-life care, examining outcomes such as in-hospital death and emergency department admissions.^4,5^ Few studies have examined whether financial resources are associated with well-being at the end of life. We hypothesized that financial resources may substantially influence well-being as they enable patients and families to access services and better medical care to address symptoms, personal care, and family and social support.

In this study, we addressed 3 gaps in the literature regarding the association between financial resources on end-of-life experiences. First, we focused on symptoms as reported by proxy respondents after a patient’s death. Pain and other symptoms are frequent at the end of life and often a source of great distress for patients and families, but we know little about the association between financial resources and symptom presence at the end of life.

Second, we measured overall wealth as opposed to income. Wealth in older age has advantages as a measure because it signifies the availability of various types of financial resources. Wealth has immediate financial value, replacing income after retirement and allowing patients to purchase and pay for necessary resources. Wealth also carries practical value since resources included in the measure, such as housing and transportation, may help facilitate or improve clinical care. Those resources may enable patients to receive treatment at their residence or provide them the means to attend their appointments. In addition, there is a cultural value associated with wealth, potentially influencing access to and the timing of treatment.^6^ For example, individuals with higher wealth may receive more attentive and comprehensive care from health care professionals, who may perceive these patients as more capable of adhering to treatment plans and affording necessary medical interventions. This preferential treatment may lead to more timely diagnoses, better management of symptoms, and overall improved health outcomes.

Third, we investigated the potential mediating pathways through which wealth may influence the presence of symptoms, including chronic conditions, functional status, and dementia status, as individuals with lower wealth tend to experience higher rates of multimorbidity, functional impairments, and dementia.^7^ These nonmutually exclusive conditions necessitate additional caregiving, medical, and social services. However, individuals with lower wealth may have more limited access to both needed paid and unpaid caregiving and other essential services.

In this study, we examined the association of wealth with the end-of-life experience of a national sample of US older adults. Our 3 objectives were as follows: (1) to evaluate the prevalence of 12 individual end-of-life symptoms (eg, pain, depression, difficulty breathing) by wealth categories; (2) to examine the association between wealth and a composite measure of high symptom burden (ie, presence of ≥7 individual end-of-life symptoms); and (3) to examine whether the association between wealth and high symptom burden at the end of life is mediated by multimorbidity, functional impairment, or dementia.

## Methods

### Study Sample

In this cohort study, we used data from the Health and Retirement Study (HRS),^8^ a nationally representative, longitudinal survey of approximately 45 000 community-dwelling adults aged 51 years or older. The publicly available dataset includes core interviews conducted every 2 years on participant health, financial, and sociodemographic characteristics. After a participant dies, a proxy respondent completes an exit interview about the end-of-life experience. This study was approved by the University of California, San Francisco Institutional Review Board, with informed consent exemptions made because the study team had no contact with the participants and not permitted to access participants’ contact information. This report adhered to the Strengthening the Reporting of Observational Studies in Epidemiology (STROBE) reporting guideline.

We used exit interviews from January 1, 2000, through February 28, 2021 (eg, for participants who died in 2000 or later), and included 9991 decedents who had an HRS core interview within 24 months before death at age 65 years or older. We excluded 482 participants (5%) whose proxy respondent did not complete the exit interview and 533 (6%) with incomplete data on end-of-life symptoms in the exit interview. Missingness resulted from HRS data collection errors in 2002 and 2020.^9,10^

### Measures

We measured wealth using participants’ self-report of assets and debt in their last core HRS interview before death. We calculated wealth as the sum of all asset components minus all debt components. Asset components included primary and secondary residences; real estate; vehicles; businesses; individual retirement accounts and Keogh accounts; stocks, mutual funds, and investments funds; checking, savings, and money market accounts; certificate of deposit accounts; government savings bonds and treasury bills; bonds and bond funds; and all other savings. Debt components included mortgages and land contracts, other home loans, and all other debt. Where an asset or debt component was missing, we used values imputed by the RAND Center for the Study of Aging, described elsewhere.^11^ We included wealth in the analysis as a 3-level categorical variable to facilitate interpretation: low wealth (bottom quartile, <$6000), medium wealth (middle 2 quartiles, $6000-$120 000), and high wealth (top quartile, >$120 000). We conducted additional analyses using wealth divided into 11 finer categories: participants in debt; participants with zero wealth; and 9 additional, equally-sized groups. The findings were consistent with our primary results using the 3-level categorization, with symptom rates decreasing steadily as wealth increased (eFigure 1 in Supplement 1).

We measured end-of-life symptom presence using proxy responses to the following exit interview question: “Was there a period of at least 1 month during the last year of [the decedent’s] life when he/she had [symptom]?” The 12 symptoms were difficulty breathing, very little appetite, frequent vomiting, difficulty controlling arms and legs, depression, periodic confusion, severe fatigue or exhaustion, difficulty being aroused or wakened or loss of consciousness, persistent wheezing or cough, uncontrolled outbursts or temper, loss of control of bowel or bladder, and often troubled with pain. We defined high symptom burden as the presence of 7 or more symptoms, ie, the top quartile of symptom burden in the sample. Measures of symptom severity, duration, or treatment or cause of death are not captured in HRS public use data and, therefore, not included in our analysis.

Sociodemographic characteristics included self-reported age, sex, marital status, race and ethnicity (Black, Hispanic, White, other group [American Indian, Alaska Native, Native Hawaiian, Pacific Islander, or any other categories not classified as Hispanic, non-Hispanic Black, or non-Hispanic White]), college education, and childhood socioeconomic status.^12^ We defined multimorbidity as the presence of 3 or more self-reported chronic conditions, including diabetes, heart disease, lung disease, cancer, stroke, arthritis, and vision and hearing impairment. Functional status was measured using activities of daily living (bathing, dressing, transferring, toileting, eating, walking across a room), and functional impairment was defined as needing help in 3 or more activities of daily living. Dementia status was assessed using the validated Langa-Weir methodology.^13^

### Statistical Analysis

We analyzed the distribution of all variables using counts and percentages, means with standard deviations, or medians with interquartile ranges. For our first objective, we evaluated the unadjusted and adjusted prevalence of all individual end-of-life symptoms by wealth and assessed whether the differences by wealth were statistically significant using χ^2^ tests. The adjusted estimated probabilities accounted for age, sex, marital status, race and ethnicity, college education, childhood socioeconomic status, proxy respondent relationship, location of death, time between death and exit interview, year of death, and having private insurance in addition to Medicare.

For our second objective, we examined the association between high symptom burden and wealth. We used multivariable, modified Poisson regression to assess whether wealth was independently associated with high symptom burden after adjusting for age, sex, marital status, race and ethnicity, college education, childhood socioeconomic status, proxy relationship, location of death, time between death and exit interview, year of death, and having private insurance in addition to Medicare. The modified Poisson regression analysis used robust error variance to estimate relative risks (RRs), which are more interpretable than odds ratios for nonrare outcomes.^14^

**Figure 1.**
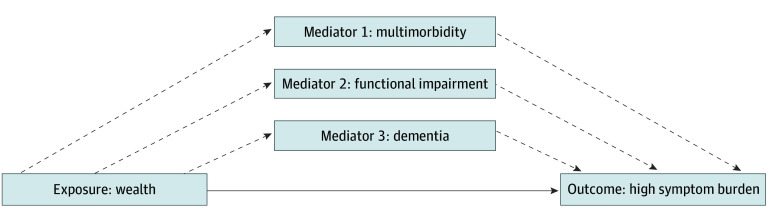
Directed Acyclic Graph Illustrating Hypothesized Pathways Among Wealth, Mediators, and End-of-Life Symptom Burden The graph highlights the role of mediators and potential confounding variables included in the analysis. Confounding variables considered were age, sex, race and ethnicity, marital status, college education, childhood socioeconomic status, proxy respondent relationship, private insurance, place of death, time between death and exit interview, and year of death. The solid arrow indicates direct associations, and dashed arrows indicate potential indirect pathways.

For our third objective, we examined potential mediators between wealth and symptom burden. First, we compared the distributions of the 3 potential mediators (multimorbidity, functional impairment, dementia) across wealth categories using χ^2^ tests. Then, we performed all analyses on complete cases, as missingness in mediators and confounders was minimal (<3%). Next, we used generalized structural equation modeling to test mediation effects, decomposing the total effect of wealth on high symptoms burden into direct and indirect pathways via multimorbidity, functional impairment, and dementia. The model specified wealth as a 3-level categorical variable and mediators and outcome as binary variables. We used modified Poisson regression with robust standard errors to estimate relative risks. We also included all confounders described in our second objective as they potentially influence the association among wealth, mediators, and outcome. Key assumptions included no unmeasured confounding, temporal ordering (ie, wealth preceding the mediators), and correct model specification. Latent variables were not used as all variables were directly measured. Our primary hypothesis was that lower wealth would be associated with higher multimorbidity, greater functional impairment, and dementia, which in turn would be associated with higher symptom burden. We described the extent of mediation as the proportion of the total effect of an exposure mediated by a specific indirect effect. Results are presented as relative risks and as coefficients from modified Poisson regression. The model is described as a directed acyclic graph in Figure 1.

We conducted a sensitivity analysis to address the possibility that certain causes of death and chronic conditions were associated with wealth and influence end-of-life symptoms. We fit a series of modified Poisson regression models, adjusting for each chronic condition individually and adjusting for all conditions together, to investigate potential confounding of wealth and high symptom burden by chronic conditions.

Analyses were performed from October 6, 2023, through November 26, 2024, using Stata, version 18.1 (StataCorp LLC) and SAS, version 9.4 (SAS Institute Inc). All analyses accounted for HRS survey weights and complex survey design.^15^ Statistical significance was set at a 2-sided *P* < .05.

## Results

The final sample included 8976 decedents (mean [SD] age, 81.3 [8.6] years; 4049 men [44.9%] and 4927 women [55.1%]; and 1287 of Black [9.5%], 624 of Hispanic [5.5%], 6911 of White [83.2%], and 154 of other [1.8%] race and ethnicity) (Table 1). A total of 3409 decedents (38.1%) were married at the last HRS core interview before death, and 1240 (15.6%) had a college education or higher. Overall, 5790 decedents (64.4%) experienced multimorbidity at the end of life, 2424 (25.6%) experienced functional impairment, and 3319 (34.4%) experienced dementia. The median time between death and proxy exit interview was 13 months (IQR, 7-19 months).

**Table 1.** Descriptive Characteristics of the Cohort by Wealth Category at the HRS Interview Before Death

Characteristic	HRS participants, No. (%)				*P* value
	Low wealth (n = 2197)	Medium wealth (n = 4534)	High wealth (n = 2245)	Total (N = 8976)	
Age, mean (SD), y	81.7 (9.2)	81.1 (8.5)	81.3 (8.2)	81.3 (8.6)	.26
Sex					
Female	1521 (69.3)	2405 (53.8)	1001 (45.8)	4927 (55.1)	<.001
Male	676 (30.7)	2129 (46.2)	1244 (54.2)	4049 (44.9)	
Race and ethnicity					
Black	570 (18.6)	636 (9.4)	81 (2.1)	1287 (9.5)	<.001
Hispanic	254 (9.5)	307 (5.4)	63 (2.3)	624 (5.5)	
White	1310 (68.8)	3520 (83.5)	2081 (94.7)	6911 (83.2)	
Other^a^	63 (3.1)	71 (1.6)	20 (1.0)	154 (1.8)	
Married or partnered	316 (13.3)	1846 (40.2)	1247 (54.7)	3409 (38.1)	<.001
Lived in nursing home	754 (38.8)	570 (13.2)	224 (11.2)	1548 (18.5)	<.001
College education or higher	111 (6.0)	440 (10.8)	689 (32.3)	1240 (15.6)	<.001
Private insurance	489 (24.2)	2101 (48.4)	1361 (61.0)	3951 (46.4)	<.001
Place of death					
Home	446 (20.5)	1351 (29.2)	776 (35.1)	2573 (28.8)	<.001
Hospital	715 (31.0)	1546 (34.2)	694 (31.0)	2955 (32.6)	
Other	1035 (48.5)	1630 (36.6)	772 (34.0)	3437 (38.6)	
Time between death and proxy exit interview, median (IQR), mo	13 (7-19)	12 (7-19)	12 (7-19)	13 (7-19)	.06
Proxy relationship to decedents					
Spouse	250 (10.8)	1448 (31.8)	1013 (45.0)	2711 (30.6)	<.001
Child	1293 (59.3)	2256 (49.4)	931 (40.9)	4480 (49.4)	
Other^b^	654 (29.9)	830 (18.8)	301 (14.1)	1785 (20.0)	
Cognition					
No impairment	433 (22.6)	1689 (39.7)	1120 (51.3)	3242 (39.0)	<.001
CIND	550 (25.7)	1317 (28.4)	548 (24.2)	2415 (26.6)	
Dementia	1214 (51.7)	1528 (32.0)	577 (24.5)	3319 (34.4)	
Comorbidities					
Diabetes	768 (34.6)	1390 (30.5)	546 (25.1)	2704 (30.0)	<.001
Cancer	500 (23.9)	1283 (28.4)	812 (37.0)	2595 (29.7)	<.001
Lung disease	534 (26.7)	1020 (22.7)	434 (20.0)	1988 (22.9)	<.001
Heart disease	1185 (55.8)	2393 (52.6)	1099 (47.9)	4677 (52.0)	<.001
Stroke	745 (33.0)	1158 (24.9)	504 (22.0)	2407 (25.9)	<.001
Arthritis	1739 (79.6)	3386 (74.5)	1530 (68.1)	6655 (73.9)	<.001
Vision impairment	1223 (54.3)	1908 (40.8)	719 (31.4)	3850 (41.3)	<.001
Hearing impairment	982 (44.8)	1819 (39.9)	804 (34.9)	3605 (39.6)	<.001
Multimorbidity^c^	1571 (73.0)	2904 (63.6)	1315 (58.6)	5790 (64.4)	<.001
Functional disability					
Activities of daily living					
Bathing	1211 (52.7)	1445 (31.0)	603 (26.4)	3259 (34.6)	<.001
Transferring from or to bed	818 (35.3)	829 (17.6)	381 (16.9)	2028 (21.4)	<.001
Dressing	1041 (44.5)	1305 (27.7)	583 (25.6)	2929 (30.9)	<.001
Eating	633 (26.7)	733 (15.2)	332 (14.3)	1698 (17.5)	<.001
Walking across room	814 (35.4)	926 (20.1)	417 (18.2)	2157 (23.0)	<.001
Toileting	692 (29.8)	696 (15.0)	312 (13.6)	1700 (17.9)	<.001
Functional impairment^d^	944 (40.4)	1025 (22.0)	455 (20.0)	2424 (25.6)	<.001
Instrumental activities of daily living					
Preparing meals	477 (29.3)	910 (21.6)	383 (19.2)	1770 (22.3)	<.001
Taking medications	306 (15.9)	536 (12.5)	234 (11.5)	1076 (12.9)	<.001
Managing finances	1076 (47.3)	1397 (30.1)	606 (26.3)	3079 (32.9)	<.001
Using the phone	635 (27.4)	854 (17.7)	383 (17.0)	1872 (19.7)	<.001
Grocery shopping	586 (39.0)	1190 (28.9)	470 (24.2)	2246 (29.4)	<.001

Abbreviations: CIND, cognitive impairment with no dementia; HRS, Health and Retirement Study.

^a^ Other races and ethnicities included American Indian, Alaska Native, Native Hawaiian, Pacific Islander, or any other categories not classified as Hispanic, non-Hispanic Black, or non-Hispanic White. These categories were collapsed into 1 group due to small sample sizes.

^b^ Other proxy relationships included spouse of a child, grandchild, brother or sister, other relative, and professional.

^c^ Multimorbidity was defined as the presence of 3 or more comorbidities.

^d^ Functional impairment was defined as needing help in 3 or more activities of daily living.

**Figure 2.**
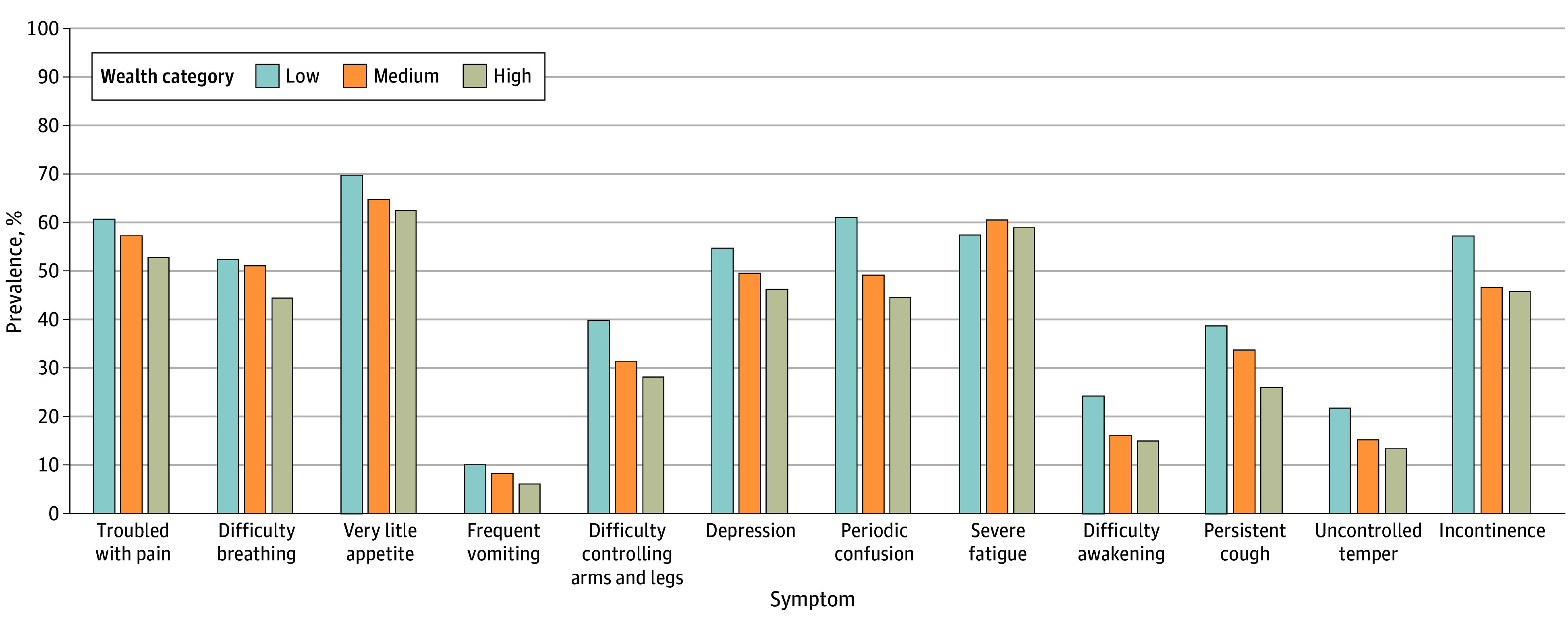
Prevalence of Individual End-of-Life Symptoms by Wealth Category All differences were statistically significant between low and medium wealth and low and high wealth except difficulty breathing and frequent vomiting between decedents with low and medium wealth and severe fatigue between decedents with low and high wealth. All unadjusted and adjusted prevalence values and corresponding *P* values are presented in eTables 1 and 2 in Supplement 1.

A total of 2197 decedents (22.5%) were included in the low wealth category, 4534 (50.5%) in the medium wealth category, and 2245 (27.1%) in the high wealth category. Ten of 12 end-of-life symptoms were significantly more prevalent in decedents with lower wealth than in decedents with medium or high wealth, including very little appetite (low wealth, 1502 [70.1%]; medium wealth, 2964 [65.1%]; high wealth, 1411 [63.0%]; low vs medium *P* = .002; low vs high *P* < .001), frequent vomiting (low wealth, 221 [10.4%]; medium wealth, 397 [8.4%]; high wealth, 759 [8.3%]; low vs medium *P* = .046; low vs high *P* < .001), difficulty controlling arms and legs (low wealth, 899 [40.2%]; medium wealth, 1462 [31.7%]; high wealth, 2996 [32.7%]; low vs medium *P* < .001; low vs high *P* < .001), depression (low wealth, 1122 [57.8%]; medium wealth, 2169 [49.5%]; high wealth, 996 [46.6%]; low vs medium *P* < .001; low vs high *P* < .001), periodic confusion (low wealth, 1333 [61.5%]; medium wealth, 2268 [49.5%]; high wealth, 1014 [45.0%]; low vs medium *P* < .001; low vs high *P* < .001), difficulty being aroused or wakened or loss of consciousness (low wealth, 529 [24.5%]; medium wealth, 755 [16.4%]; high wealth, 342 [15.2%]; low vs medium *P* < .001; low vs high *P* < .001), persistent wheezing or cough (low wealth, 837 [38.9%]; medium wealth, 1539 [34.1%]; high wealth, 589 [26.3%]; low vs medium *P* = .007; low vs high *P* < .001), uncontrolled outbursts or temper (low wealth, 466 [22.0%]; medium wealth, 737 [15.3%]; high wealth, 305 [13.7%]; low vs medium *P* < .001; low vs high *P* < .001), loss of control of bowel or bladder (low wealth, 1227 [57.5%]; medium wealth, 2095 [46.9%]; high wealth, 1029 [46.2%]; low vs medium *P* < .001; low vs high *P* < .001), and often troubled with pain (low wealth, 1298 [61.1%]; medium wealth, 2573 [57.5%]; high wealth, 1149 [53.1%]; low vs medium *P* = .02; low vs high *P* < .001). Fatigue and difficulty breathing had more complicated patterns across wealth categories (Figure 2; eTables 1 and 2 in Supplement 1).

High symptom burden (ie, ≥7 symptoms) was significantly associated with wealth, ie, less burden with greater wealth (low wealth, 800 decedents [37.0%]; medium wealth, 1301 decedents [28.0%]; high wealth, 512 decedents [23.2%]; *P* < .001). Significant associations between lower wealth and high symptom burden persisted after adjusting for age, sex, marital status, race and ethnicity, college education, childhood socioeconomic status, proxy respondent relationship, private insurance, place of death, time between death and exit interview, and year of death compared with the medium and high wealth groups (adjusted RR [ARR], 0.79 [95% CI, 0.73-0.87] and 0.71 [95% CI, 0.63-0.79], respectively) (Table 2).

**Table 2.** Risk of High Symptom Burden, Multimorbidity, Functional Impairment, and Dementia by Wealth Category

Outcome and wealth category	Prevalence, %	Unadjusted RR (95% CI)	*P *value	Adjusted RR (95% CI)^a^	*P *value
**High symptom burden**					
Low wealth	37.0	1 [Reference]	NA	1 [Reference]	NA
Medium wealth	28.0	0.76 (0.70-0.82)	<.001	0.79 (0.73-0.87)	<.001
High wealth	23.3	0.63 (0.57-0.69)	<.001	0.71 (0.63-0.79)	<.001
**Multimorbidity**					
Low wealth	73.0	1 [Reference]	NA	1 [Reference]	NA
Medium wealth	63.6	0.87 (0.83-0.91)	<.001	0.87 (0.83-0.91)	<.001
High wealth	58.6	0.8 (0.76-0.85)	<.001	0.83 (0.78-0.88)	<.001
**Functional impairment**					
Low wealth	40.4	1 [Reference]	NA	1 [Reference]	NA
Medium wealth	22.0	0.54 (0.50-0.6)	<.001	0.67 (0.61-0.72)	<.001
High wealth	20.1	0.5 (0.44-0.56)	<.001	0.65 (0.57-0.74)	<.001
**Dementia**					
Low wealth	51.7	1 [Reference]	NA	1 [Reference]	NA
Medium wealth	32.0	0.62 (0.58-0.66)	<.001	0.75 (0.70-0.80)	<.001
High wealth	24.5	0.47 (0.43-0.52)	<.001	0.64 (0.58-0.71)	<.001

Abbreviations: NA, not applicable; RR, relative risk.

^a^ Adjusted for age, sex, marital status, race and ethnicity, college education, childhood socioeconomic status, proxy respondent relationship, private insurance, place of death, time between death and exit interview, and year of death.

Decedents with lower wealth had higher rates of multimorbidity, functional impairment, and dementia than those with medium or high wealth. Specifically, 1571 decedents (73.0%) in the low wealth group experienced multimorbidity vs 2904 (63.6%) in the medium and 1315 (58.6%) in the high wealth groups (*P* < .001). We observed functional impairment in 944 decedents (40.4%) in the low wealth group vs 1025 (22.0%) in the medium and 455 (20.0%) in the high wealth groups (*P* < .001). Additionally, 1214 decedents (51.7%) in the low wealth group had dementia vs 1528 (32.0%) in the medium and 577 (24.5%) in the high wealth groups (*P* < .001) (Table 1). Associations between these hypothesized mediators and wealth remained significant in the adjusted generalized structural equation modeling: multimorbidity (low vs medium wealth: ARR, 0.87 [95% CI, 0.83-0.91]; low vs high wealth: ARR, 0.83 [95% CI, 0.78-0.88]), function (low vs medium wealth: ARR, 0.67 [95% CI, 0.61-0.72]; low vs high wealth: ARR, 0.65 [95% CI, 0.57-0.73]), and dementia (low vs medium wealth: ARR, 0.75 [95% CI, 0.70-.079]; low vs high wealth, ARR, 0.64 [95% CI, 0.58-0.71]) (Table 3). Multimorbidity, functional impairment, and dementia were all additionally associated with end-of-life symptom burden (low vs medium wealth: ARR, 0.88 [95% CI, 0.80-0.95]; low vs high wealth: ARR, 0.80 [95% CI, 0.71-0.89]).

**Table 3.** Direct and Indirect Associations of Wealth Categories With End-of-Life High Symptom Burden

Association and hypothesized mediator	Adjusted RR (95% CI)^a^^,^^b^			
	Multimorbidity	Functional impairment	Dementia	End-of-life symptom burden
Direct				
Low vs medium wealth	0.87 (0.83-0.91)	0.67 (0.61-0.72)	0.75 (0.70-0.79)	0.88 (0.80-0.95)
Low vs high wealth	0.83 (0.78-0.88)	0.65 (0.57-0.73)	0.64 (0.58-0.71)	0.80 (0.71-0.89)
Indirect^c^				
Via multimorbidity				
Low vs medium wealth	NA	NA	NA	0.95 (0.93-0.97)
Low vs high wealth	NA	NA	NA	0.93 (0.90-0.96)
Via functional impairment				
Low vs medium wealth	NA	NA	NA	0.87 (0.82-0.91)
Low vs high wealth	NA	NA	NA	0.86 (0.80-0.91)
Via dementia				
Low vs medium wealth	NA	NA	NA	0.98 (0.95-1.00)
Low vs high wealth	NA	NA	NA	0.96 (0.93-1.00)
Total				
Low vs medium wealth	NA	NA	NA	0.70 (0.64-0.77)
Low vs high wealth	NA	NA	NA	0.61 (0.53-0.69)
Direct by mediator				
Multimorbidity	NA	NA	NA	1.48 (1.31-1.65)
Functional impairment	NA	NA	NA	1.42 (1.28-1.57)
Dementia	NA	NA	NA	1.09 (0.99-1.18)

Abbreviations: NA, not applicable; RR, relative risk.

^a^ Adjusted for age, sex, marital status, race and ethnicity, college education, childhood socioeconomic status, proxy respondent relationship, private insurance, place of death, time between death and exit interview, and year of death.

^b^ Regression coefficients are presented in eTable 3 in Supplement 1.

^c^ The indirect associations were measured through 3 hypothesized mediators (multimorbidity, functional impairment, and dementia).

All 3 mediators significantly mediated the association of wealth with end-of-life symptom burden, with functional impairment accounting for most of the effect between medium and low wealth (coefficient, −0.41; 95% CI, −0.48 to −0.33) and between high and low wealth (coefficient, −0.43; 95% CI, −0.56 to −0.30) (eTable 3 in Supplement 1). The direct association of wealth on end-of-life symptom burden remained significant even after accounting for indirect associations through the 3 mediators (multimorbidity: coefficient, 0.39 [95% CI, 0.27-0.51]; functional impairment: coefficient, 0.35 [95% CI, 0.25-0.45]; dementia: coefficient, 0.08 [95% CI, −0.01 to 0.17]). In sensitivity analysis controlling for individual chronic conditions, the association of wealth with high symptom burden was attenuated but remained significant (eTable 4 in Supplement 1).

## Discussion

This nationally representative cohort study found that low wealth was associated with greater symptom burden at the end of life. Compared with decedents with middle and high wealth, those with low wealth had a higher prevalence of most end-of-life symptoms. While differences in some individual symptoms seemed small, these differences cumulatively translated into a substantial population burden of symptoms, as reflected by the population attributable fraction.^16^ Specifically, wealth disparities accounted for 12% of the symptom burden between the low and medium wealth groups and 20% between the low and high wealth groups. While this contribution is lower than that of other factors, such as comorbidities (population attributable factor, 39%) or functional impairment (population attributable factor, 35%), it is still substantial. Decedents with low wealth were 26% more likely to experience high symptom burden at the end of life than those with medium wealth and 42% more likely to experience high symptom burden than those with high wealth. Wealth, therefore, may play a substantial, and potentially underappreciated, role in a negative end-of-life experience.

We also found that multimorbidity, functional impairment, and dementia were all associated with both wealth and high symptom burden at the end of life. The 3 conditions mediated the association between wealth and high symptom burden, with functional impairment accounting for at least one-third of the total effect of wealth. This finding suggests that decedents with low wealth may have a high symptom burden in part because they have more chronic conditions and more functional impairment at the end of life.

Previous research has consistently identified associations between low socioeconomic status and end-of-life outcomes considered to be of poorer quality (ie, more acute medical care use,^17^ increased likelihood of dying in institutional settings,^4,5,18^ lower likelihood of using hospice^19^). Our results align with these well-documented patterns and expand to the patient-centered outcome of symptom burden at the end of life. Patients with multimorbidity, functional impairment, and dementia require more assistance and caregiving, which may be more difficult to obtain with limited financial resources. Older adults may face adverse consequences from unmet needs for assistance with self-care, household tasks, and mobility, which have been reported to be more prevalent among individuals dually eligible for Medicare and Medicaid who have more limited financial resources.^20^ Similarly, among home hospice patients, those with lower income have been reported to be less likely to remain at home until death, suggesting a need for intense support to overcome financial resource disparities.^5^

Patients with lower wealth may be doubly challenged at the end of life if they experience a great need for support due to multimorbidity, functional impairment, and end-of-life symptoms as they may lack the resources to address such needs. We hypothesize several reasons why these disparities may exist. Individuals with very low resources may have a harder time getting adequate support for the same disease or symptom burden (eg, paid caregivers) as those with more resources. They may not want or be able to remain at home but, eg, may have difficulty paying utilities or lack stable housing. Additionally, certain health conditions can exacerbate financial difficulties over time (eg, dementia).^21^ Given the cumulative burden of having very low resources over their life course,^18^ individuals with low wealth may experience a different clinical trajectory than their counterparts with more resources.

Addressing these systemic issues with targeted health policies and programs is crucial for improving the end-of-life experience for older adults with backgrounds of limited resources. The dominant model for end-of-life care in the US is the Medicare Hospice Benefit, which delivers end-of-life care to 52% of Medicare beneficiaries.^22^ Though we did not examine the use of hospice, our findings might inform efforts to modernize the benefit. Policies that respond to the needs of patients with limited financial resources, such as a low-income add-on hospice benefit that provides additional functional supports, may mitigate financial disparities at the end of life and lead to a more equitable end-of-life experience across diverse patient populations. Wealth disparities in symptom burden may also be influenced by access to supportive services, such as Medicaid, which our study was not designed to evaluate. Future work to account for complex eligibility criteria, including health, socioeconomic factors, and state of residence, could examine how Medicaid and similar programs influence symptom burden among populations with low wealth.

### Limitations

Despite using a nationally representative survey, this study has several limitations. First, we did not have access to cause-of-death data. Some causes of death are associated with both wealth and high symptom burden; not accounting for these may lead to underestimating direct associations of wealth on symptom burden. We performed a sensitivity analysis to account for individual diagnoses before death, including cancer, and found that the association of wealth with symptom burden remained significant (eTable 4 in Supplement 1). Second, proxy recall bias may influence exit interview responses; however, the HRS stated that exit interview proxy respondents were “knowledgeable about the health, family, and financial situation of the deceased”^10^^(p1)^ and the majority (90%) of interviews occurred within 2 years of the decedent’s death.^10^ Third, we could not examine the direction of the wealth and symptom association due to not having data on duration of illness or use of caregivers (ie, a long illness might have depleted wealth). Finally, we assessed the presence of end-of-life symptoms as binary outcomes (yes or no) in the absence of information in HRS about severity, duration, or impact. Future analyses using data sources that include these measures could provide a more nuanced understanding of the association between wealth and end-of-life symptom experiences.

## Conclusions

This cohort study found evidence of wealth inequalities in the end-of-life experiences of older adults. Our findings suggest that individuals with lower wealth may experience high end-of-life symptom burden that may be partly attributed to worse functional status. Addressing functional impairment may be an important consideration for end-of-life policies and programs aimed at improving care for patients with limited financial resources.

## Notes

Supplement 1. **eTable 1.** Unadjusted Prevalence of Individual End-of-Life Symptoms by Wealth Category **eTable 2.** Adjusted Prevalence and 95% CIs of Individual End-of-Life Symptoms by Wealth Category **eTable 3.** Regression Coefficients for Direct and Indirect Associations of Wealth Categories on End-of-Life High Symptom Burden **eTable 4.** Adjusted Relative Risks (RRs) of High Symptom Burden by Wealth Category, Including Adjustment for Comorbidities **eFigure 1.** Prevalence of Individual End-of-Life Symptoms and Overall Symptom Burden Across 11 Wealth Categories **eFigure 2.** Prevalence of High Symptom Burden Across 3 Wealth Categories (Low, Medium, High) Using Different Symptom Burden Cutoffs

Supplement 2.
Data Sharing Statement
