# Supplementary material for: Wealth Disparities in End-of-Life Symptom Burden Among Older Adults
Source: JAMA Netw Open. 2025 Mar 6;8(3):e250201. doi: 10.1001/jamanetworkopen.2025.0201 (PMC11886723; doi:10.1001/jamanetworkopen.2025.0201)
Supplement: Supplement 1. — eTable 1. Unadjusted Prevalence of Individual End-of-Life Symptoms by Wealth Category eTable 2. Adjusted Prevalence and 95% CIs of Individual End-of-Life Symptoms by Wealth Category eTable 3. Regression Coefficients for Direct and Indirect Associations of Wealth Categories on End-of-Life High Symptom Burden eTable 4. Adjusted Relative Risks (RRs) of High Symptom Burden by Wealth Category, Including Adjustment for Comorbidities eFigure 1. Prevalence of Individual End-of-Life Symptoms and Overall Symptom Burden Across 11 Wealth Categories eFigure 2. Prevalence of High Symptom Burden Across 3 Wealth Categories (Low, Medium, High) Using Different Symptom Burden Cutoffs [file jamanetwopen-e250201-s001.pdf]

Supplemental Online Content

Cenzer I, Covinsky KE, Cross SH, et al. Wealth disparities in end-of-life symptom burden among older adults. *JAMA Netw Open*. 2025;8(3):e250201.
doi:10.1001/jamanetworkopen.2025.0201

eTable 1. Unadjusted Prevalence of Individual End-of-Life Symptoms by Wealth Category

eTable 2. Adjusted Prevalence and 95% CIs of Individual End-of-Life Symptoms by Wealth Category

eTable 3. Regression Coefficients for Direct and Indirect Associations of Wealth Categories on End-of-Life High Symptom Burden

eTable 4. Adjusted Relative Risks (RRs) of High Symptom Burden by Wealth Category, Including Adjustment for Comorbidities

eFigure 1. Prevalence of Individual End-of-Life Symptoms and Overall Symptom Burden Across 11 Wealth Categories

eFigure 2. Prevalence of High Symptom Burden Across 3 Wealth Categories (Low, Medium, High) Using Different Symptom Burden Cutoffs

This supplemental material has been provided by the authors to give readers additional information about their work.

© 2025 Cenzer I et al. *JAMA Network Open*.

eTable 1. Unadjusted Prevalence of Individual End-of-Life Symptoms by Wealth Category

	Wealth					
**End-of-life symptom**	**Low Wealth**	**Medium Wealth**	**High Wealth**	**Total**	**p-value (low-medium wealth)**	**p-value (low-high wealth)**
Troubled with pain	1,298 (61.1%)	2,573 (57.5%)	1,149 (53.1%)	5,020 (57.1%)	0.02	<0.001
Difficulty breathing	1,125 (52.7%)	2,336 (51.4%)	998 (44.9%)	4,459 (49.9%)	0.41	<0.001
Very little appetite	1,502 (70.1%)	2,964 (65.1%)	1,411 (63.0%)	5,877 (65.7%)	0.002	<0.001
Frequent vomiting	221 (10.4%)	397 (8.4%)	141 (6.3%)	759 (8.3%)	0.05	<0.001
Difficulty controlling arms/legs	899 (40.2%)	1,462 (31.7%)	635 (28.3%)	2,996 (32.7%)	<0.001	<0.001
Depression	1,122 (55.0%)	2,169 (49.7%)	996 (46.6%)	4,287 (50.1%)	<0.001	<0.001
Periodic confusion	1,333 (61.5%)	2,268 (49.5%)	1,014 (45.0%)	4,615 (51.0%)	<0.001	<0.001
Severe fatigue	1,221 (57.8%)	2,704 (60.8%)	1,314 (59.3%)	5,239 (59.7%)	0.04	0.44
Difficulty awakening	529 (24.5%)	755 (16.4%)	342 (15.2%)	1,626 (17.9%)	<0.001	<0.001
Persistent cough	837 (38.9%)	1,539 (34.1%)	589 (26.3%)	2,965 (33.1%)	0.007	<0.001
Uncontrolled temper	466 (22.0%)	737 (15.3%)	305 (13.7%)	1,508 (16.4%)	<0.001	<0.001
Incontinence	1,227 (57.5%)	2,095 (46.9%)	1,029 (46.2%)	4,351 (49.0%)	<0.001	<0.001
High Symptom Burden	800 (37.0%)	1,301 (28.0%)	512 (23.3%)	2,613 (28.7%)	<0.001	<0.001

© 2025 Cenzer I et al. *JAMA Network Open*.

eTable 2. Adjusted Prevalence and 95% CIs of Individual End-of-Life Symptoms by Wealth Category

The results are adjusted for age, gender, marital status, self-reported race/ethnicity, college education, childhood SES, proxy relationship, private insurance, place of death, months between death and exit interview, and year of death.

	Wealth					
**End-of-life symptom**	**Low Wealth**	**Medium Wealth**	**High Wealth**	**Total**	**p-value (low-medium wealth)**	**p-value (low-high wealth)**
Troubled with pain	59.4% (56.3,62.5)	57% (55.4,58.7)	55.1% (52.6,57.7)	57.1% (55.7,58.4)	0.16	0.04
Difficulty breathing	53.4% (50.9,56)	51% (49.3,52.7)	45.8% (43.4,48.2)	50.1% (48.9,51.4)	0.12	<0.001
Very little appetite	67.5% (64.6,70.3)	65.3% (63.7,66.9)	64.4% (61.3,67.6)	65.6% (64.5,66.6)	0.20	0.23
Frequent vomiting	8.7% (7.1,10.3)	8.5% (7.4,9.5)	7.5% (6,9.1)	8.3% (7.6,9.1)	0.82	0.33
Difficulty controlling arms/legs	39.9% (37.6,42.3)	31.7% (29.9,33.6)	28.4% (26.1,30.7)	32.7% (31.5,33.8)	<0.001	<0.001
Depression	53.5% (50.6,56.3)	49.5% (47.7,51.3)	47.5% (45,50)	49.9% (48.4,51.4)	0.008	0.003
Periodic confusion	57.2% (54.7,59.7)	50.1% (48.4,51.8)	46.1% (43.9,48.3)	50.7% (49.4,52.1)	<0.001	<0.001
Severe fatigue	58.4% (55.6,61.2)	60.9% (59.4,62.5)	59.1% (56.2,61.9)	59.9% (58.8,61)	0.13	0.78
Difficulty awakening	22.6% (20.4,24.7)	16.3% (15,17.6)	15.5% (13.6,17.5)	17.6% (16.6,18.5)	<0.001	<0.001
Persistent cough	39.3% (36.5,42)	33.8% (32,35.6)	26.6% (24.3,29)	33.1% (31.9,34.3)	0.003	<0.001
Uncontrolled temper	21% (18.4,23.5)	15.1% (14,16.3)	14.3% (12.5,16.1)	16.3% (15.3,17.2)	<0.001	<0.001
Incontinence	54.8% (52.7,57)	47.6% (46,49.2)	46.9% (44.5,49.2)	49.1% (48,50.1)	<0.001	<0.001
High Symptom Burden	34.9% (32.2,37.6)	27.7% (26.3,29.1)	24.6% (22.3,27)	28.6% (27.3,29.8)	<0.001	<0.001

© 2025 Cenzer I et al. *JAMA Network Open*.

eTable 3. Regression Coefficients for Direct and Indirect Associations of Wealth Categories on End-of-Life High Symptom Burden

The indirect effects are measured through three hypothesized mediators (multimorbidity, functional impairment, and dementia).

	Outcome				
	**Multimorbidity**	**Function**	**Dementia**	**EOL symptom burden**	**% of total effect**
Direct Effects					
Low wealth vs. Medium wealth	-0.14 (-0.18,-0.1)	-0.41 (-0.48,-0.33)	-0.29 (-0.36,-0.23)	-0.13 (-0.22,-0.05)	37%
Low wealth vs. High wealth	-0.19 (-0.25,-0.13)	-0.43 (-0.56,-0.3)	-0.44 (-0.54,-0.34)	-0.23 (-0.34,-0.11)	46%
Indirect Effects					
Via Multimorbidity					
Low wealth vs. Medium wealth	-	-	-	-0.05 (-0.08,-0.03)	15%
Low wealth vs. High wealth	-	-	-	-0.07 (-0.1,-0.04)	15%
Via Function					
Low wealth vs. Medium wealth	-	-	-	-0.14 (-0.2,-0.09)	40%
Low wealth vs. High wealth	-	-	-	-0.15 (-0.22,-0.09)	31%
Via Dementia					
Low wealth vs. Medium wealth	-	-	-	-0.02 (-0.05,0)	7%
Low wealth vs. High wealth	-	-	-	-0.04 (-0.08,0)	7%
Total Effect					
Low wealth vs. Medium wealth	-	-	-	-0.35 (-0.45,-0.26)	
Low wealth vs. High wealth	-	-	-	-0.49 (-0.62,-0.36)	
Direct effects by mediators					
Multimorbidity	-	-	-	0.39 (0.27,0.51)	
Function	-	-	-	0.35 (0.25,0.45)	
Dementia	-	-	-	0.08 (-0.01,0.17)	

© 2025 Cenzer I et al. *JAMA Network Open*.

eTable 4. Adjusted Relative Risks (RR) of High Symptoms Burden by Wealth Category, Including Adjustment for Comorbidities

Outcome: HIGH SYMPTOMS BURDEN	Adjusted Relative Risk													
Characteristics	RR (95% CI)	p-value	RR (95% CI)	p-value	RR (95% CI)	p-value	RR (95% CI)	p-value	RR (95% CI)	p-value	RR (95% CI)	p-value	RR (95% CI)	p-value
Wealth														
Low wealth vs. Medium wealth	0.8 (0.73,0.87)	<0.001	0.8 (0.73,0.87)	<0.001	0.79 (0.72,0.86)	<0.001	0.82 (0.75,0.89)	<0.001	0.8 (0.74,0.87)	<0.001	0.81 (0.74,0.88)	<0.001	0.84 (0.77,0.91)	<0.001
Low wealth vs. High wealth	0.71 (0.64,0.8)	<0.001	0.71 (0.64,0.8)	<0.001	0.7 (0.62,0.78)	<0.001	0.74 (0.66,0.83)	<0.001	0.72 (0.65,0.81)	<0.001	0.72 (0.64,0.81)	<0.001	0.77 (0.68,0.86)	<0.001
Age [a]	0.98 (0.97,0.99)	<0.001	0.98 (0.98,0.99)	<0.001	0.98 (0.97,0.99)	<0.001	0.98 (0.98,0.99)	<0.001	0.98 (0.97,0.98)	<0.001	0.98 (0.97,0.98)	<0.001	0.98 (0.98,0.99)	<0.001
Women	1.02 (0.95,1.1)	0.56	1.03 (0.96,1.11)	0.38	1.03 (0.96,1.11)	0.41	1.04 (0.96,1.12)	0.31	1.05 (0.97,1.13)	0.23	1.03 (0.95,1.11)	0.48	1.05 (0.97,1.13)	0.21
Married/partnered	1.03 (0.89,1.18)	0.73	1.02 (0.89,1.18)	0.75	1.02 (0.88,1.18)	0.77	1.03 (0.89,1.19)	0.72	1.02 (0.89,1.18)	0.75	1.03 (0.89,1.19)	0.72	1.03 (0.89,1.18)	0.70
cSES	0.93 (0.88,0.97)	0.003	0.93 (0.89,0.97)	0.002	0.93 (0.88,0.97)	0.002	0.93 (0.89,0.98)	0.005	0.93 (0.89,0.98)	0.004	0.93 (0.88,0.97)	0.002	0.94 (0.9,0.98)	0.01
Race/Ethnicity														
White	1		1		1		1		1		1		1	
AA	0.79 (0.7,0.89)	<0.001	0.79 (0.7,0.89)	<0.001	0.8 (0.71,0.91)	0.001	0.86 (0.76,0.97)	0.01	0.81 (0.72,0.92)	0.001	0.78 (0.69,0.89)	<0.001	0.85 (0.75,0.95)	0.007
Hispanic	1.04 (0.87,1.24)	0.66	1.03 (0.86,1.22)	0.76	1.06 (0.88,1.27)	0.54	1.13 (0.94,1.36)	0.19	1.09 (0.91,1.3)	0.36	1.05 (0.88,1.25)	0.58	1.15 (0.95,1.39)	0.16
Other	1 (0.78,1.26)	0.97	0.99 (0.78,1.26)	0.95	1 (0.79,1.26)	0.99	1.01 (0.81,1.25)	0.92	1.01 (0.79,1.29)	0.96	1.01 (0.8,1.26)	0.94	1.04 (0.84,1.29)	0.71
College education	0.88 (0.76,1.01)	0.08	0.88 (0.76,1.01)	0.07	0.87 (0.75,1.01)	0.06	0.9 (0.78,1.04)	0.16	0.89 (0.77,1.03)	0.11	0.88 (0.76,1.01)	0.07	0.91 (0.79,1.06)	0.22
Proxy relationship to decedents														
Spouse	1		1		1		1		1		1		1	
Child	1.19 (1.05,1.34)	0.006	1.19 (1.05,1.34)	0.006	1.19 (1.05,1.34)	0.006	1.18 (1.04,1.33)	0.009	1.19 (1.05,1.34)	0.006	1.2 (1.06,1.35)	0.004	1.19 (1.06,1.34)	0.005
Other	1.11 (0.96,1.27)	0.15	1.11 (0.96,1.27)	0.15	1.11 (0.97,1.27)	0.14	1.1 (0.96,1.27)	0.18	1.11 (0.97,1.28)	0.13	1.12 (0.98,1.28)	0.10	1.13 (0.98,1.3)	0.08
Private insurance	0.96 (0.88,1.04)	0.32	0.96 (0.88,1.04)	0.35	0.96 (0.88,1.04)	0.29	0.97 (0.89,1.06)	0.51	0.96 (0.89,1.04)	0.35	0.97 (0.89,1.05)	0.45	0.98 (0.9,1.06)	0.60
Place of death														
Home	1		1		1		1		1		1		1	
Hospital	0.84 (0.75,0.94)	0.003	0.84 (0.75,0.94)	0.003	0.85 (0.76,0.95)	0.005	0.84 (0.75,0.95)	0.004	0.83 (0.74,0.93)	0.001	0.83 (0.75,0.93)	0.002	0.83 (0.74,0.93)	0.002
Other	1.33 (1.21,1.47)	<0.001	1.33 (1.2,1.47)	<0.001	1.34 (1.21,1.48)	<0.001	1.36 (1.23,1.5)	<0.001	1.33 (1.21,1.47)	<0.001	1.31 (1.18,1.45)	0	1.34 (1.21,1.48)	<0.001
Months between death and proxy interview	0.99 (0.98,0.99)	<0.001	0.99 (0.98,0.99)	<0.001	0.99 (0.98,0.99)	<0.001	0.99 (0.98,0.99)	<0.001	0.99 (0.98,0.99)	<0.001	0.99 (0.98,0.99)	0	0.99 (0.98,0.99)	<0.001
Year of death														
2000 to 2004	1		1		1		1		1		1		1	
2005 to 2009	1.09 (0.97,1.22)	0.15	1.09 (0.97,1.23)	0.13	1.09 (0.97,1.23)	0.13	1.09 (0.98,1.22)	0.11	1.1 (0.98,1.24)	0.11	1.11 (0.98,1.24)	0.09	1.08 (0.97,1.21)	0.14
2010 to 2014	1.18 (1.06,1.31)	0.002	1.19 (1.07,1.32)	0.002	1.18 (1.07,1.31)	0.002	1.17 (1.05,1.29)	0.004	1.2 (1.08,1.33)	0.001	1.2 (1.08,1.33)	0.001	1.13 (1.02,1.26)	0.02
2015 to 2021	1.16 (1,1.33)	0.05	1.17 (1.01,1.35)	0.04	1.17 (1.01,1.35)	0.03	1.15 (1,1.33)	0.05	1.17 (1.02,1.35)	0.03	1.19 (1.03,1.37)	0.02	1.12 (0.98,1.29)	0.09
Hypertension	1.13 (1.05,1.22)	0.002											1.07 (0.99,1.15)	0.08
Diabetes			1.12 (1.02,1.22)	0.02									1.08 (0.98,1.19)	0.11
Cancer					1.13 (1.03,1.24)	0.008							1.12 (1.03,1.23)	0.01
Lung disease							1.56 (1.43,1.7)	<0.001					1.53 (1.4,1.68)	<0.001
Heart Disease									1.25 (1.17,1.35)	<0.001			1.15 (1.07,1.24)	<0.001
Stroke											1.28 (1.17,1.39)	<0.001	1.24 (1.14,1.35)	<0.001

[a] Age is measured in years.

© 2025 Cenzer I et al. *JAMA Network Open*.

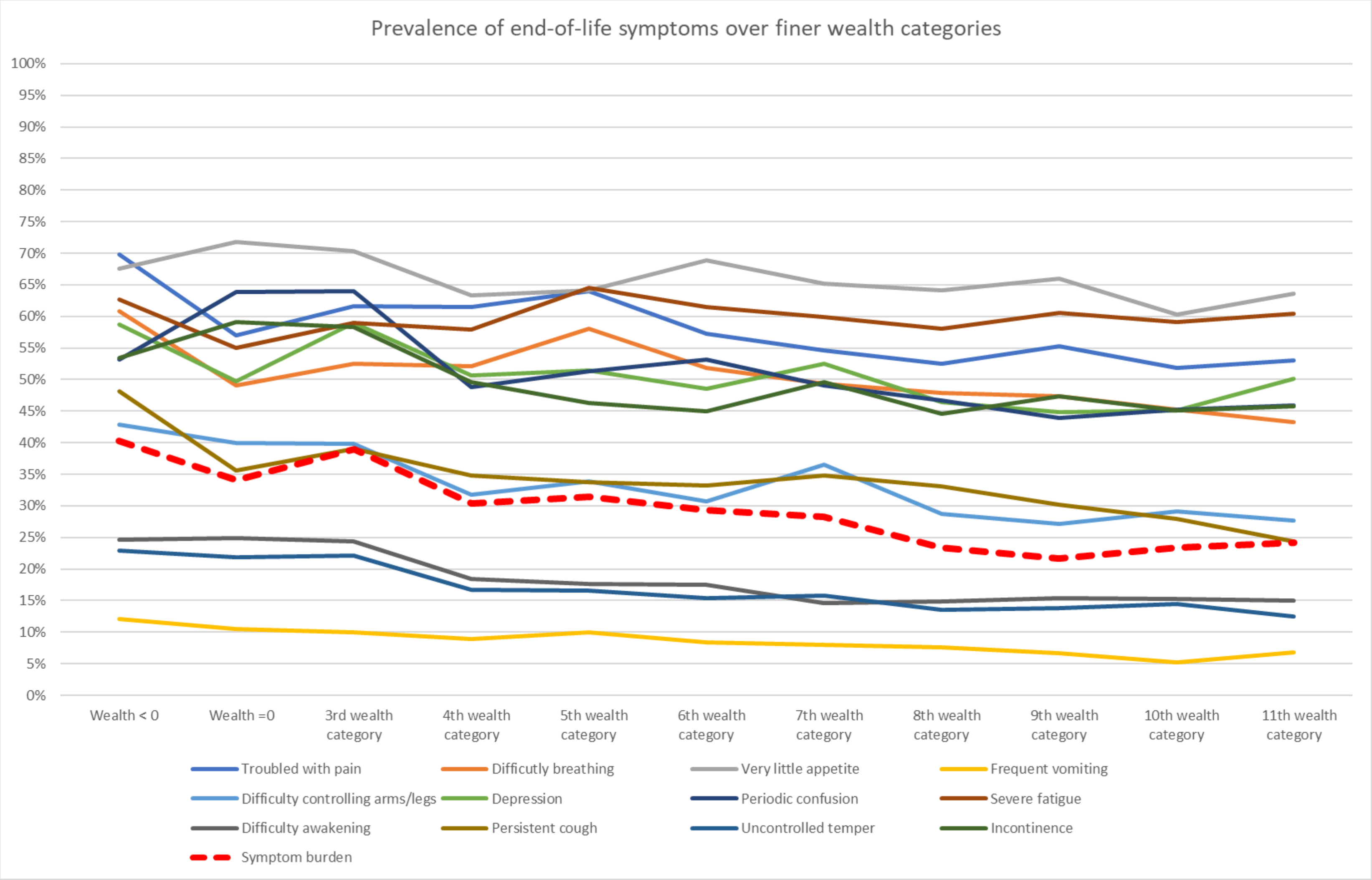

eFigure. Prevalence of Individual End-of-Life Symptoms and Overall Symptom Burden Across 11 Wealth Categories. Wealth categories include participants with debt (wealth < $0), participants with zero wealth (wealth = $0), and nine additional equally sized wealth groups. Symptom burden is represented as the proportion of individuals experiencing seven or more symptoms.

© 2025 Cenzer I et al. *JAMA Network Open*.

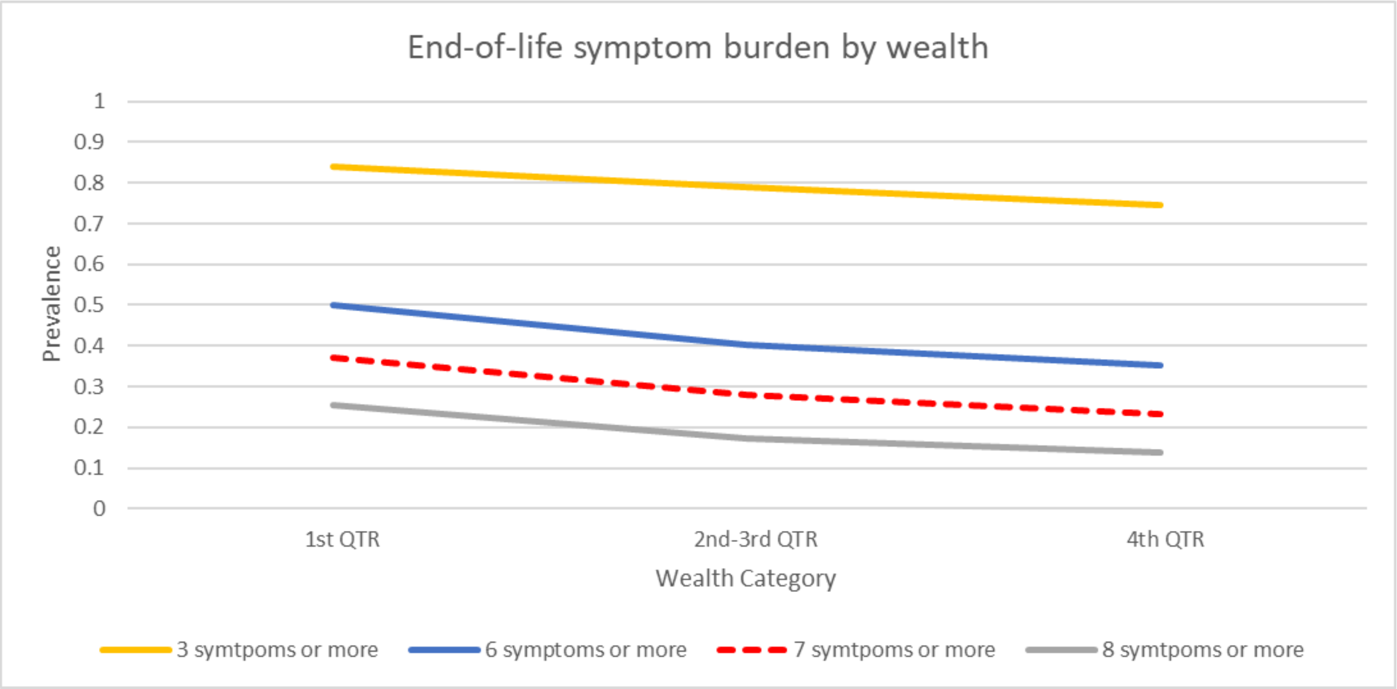

eFigure 2. Prevalence of High Symptom Burden Across 3 Wealth Categories (Low, Medium, High) Using Different Symptom Burden Cutoffs. High symptom burden is defined as experiencing 3 or more, 6 or more, 7 or more, or 8 or more symptoms.

© 2025 Cenzer I et al. *JAMA Network Open*.
