# Supplementary material for: Wealth Disparities in End-of-Life Symptom Burden Among Older Adults
Source: JAMA Netw Open. 2025 Mar 6;8(3):e250201. doi: 10.1001/jamanetworkopen.2025.0201 (PMC11886723; doi:10.1001/jamanetworkopen.2025.0201)
Supplement: Supplement 2. — Data Sharing Statement [file jamanetwopen-e250201-s002.pdf]

Data Sharing Statement

Cenzer. Wealth Disparities in End-of-Life Symptom Burden Among Older Adults. *JAMA Netw Open*. Published March 06, 2025. doi:10.1001/jamanetworkopen.2025.0201

Data
Data available: Yes
Data types: Other (please specify)
Additional Information: This is publicly available data that can be downloaded from the Health and Retirement Study website.
How to access data: This is publicly available data that can be downloaded from the Health and Retirement Study website.
When available: With publication

Supporting Documents
Document types: Other (please specify)
Additional Information: This is publicly available data that can be downloaded from the Health and Retirement Study website.
How to access documents: This is publicly available data that can be downloaded from the Health and Retirement Study website.
When available: With publication

Additional Information
Who can access the data: This is publicly available data that can be downloaded from the Health and Retirement Study website.
Types of analyses: This is publicly available data that can be downloaded from the Health and Retirement Study website.
Mechanisms of data availability: This is publicly available data that can be downloaded from the Health and Retirement Study website.
Any additional restrictions: This is publicly available data that can be downloaded from the Health and Retirement Study website.
